# On-chip optical mode conversion based on dynamic grating in photonic-phononic hybrid waveguide

**DOI:** 10.1038/srep10346

**Published:** 2015-05-21

**Authors:** Guodong Chen, Ruiwen Zhang, Junqiang Sun

**Affiliations:** 1 Wuhan National Laboratory for Optoelectronics, Huazhong University of Science and Technology, Wuhan, China

## Abstract

We present a scheme for reversible and tunable on-chip optical mode conversion based on dynamic grating in a hybrid photonic-phononic waveguide. The dynamic grating is built up through the acousto-optic effect and the theoretical model of the optical mode conversion is developed by considering the geometrical deformation and refractive index change. Three kinds of mode conversions are able to be realized using the same hybrid waveguide structure in a large bandwidth by only changing the launched acoustic frequency. The complete mode conversion can be achieved by choosing a proper acoustic power under a given waveguide length.

The acousto-optic effect is a specific case of photoelasticity, which can promote the diffraction of light by sound through a sonic grating^1^, and it has been successfully used in modulators^2^, frequency shifters^3^ and tunable filters^4^. Recently, tunable mode conversions are implemented by utilizing the acousto-optic effect in optical fibers^5^,^6^,^7^, which is useful in mode division multiplexing (MDM) over multi-mode fibers. Meanwhile, the on-chip mode converter, serving as an important device to add and drop optical signals, has also been proposed as an important component in the integrated MDM systems^8^,^9^,^10^. At present, the main methods using for on-chip mode converter are adiabatic coupling and resonant coupling. The mode conversion based on adiabatic coupling is vulnerable to impact from external environment, and cannot realize tunable mode conversion^10^,^11^,^12^. In traditional resonant coupling, an optical field is converted from one mode to another by designing the coupling region of a resonant mode converter to be half the beat length^13^. While resonant mode converter can be made very short, the difficulty is to determine the precise beat length due to device parameter variations from fabrication. With the external injection of the acoustic wave, the optical mode converters based on acousto-optic effect can relax the fabrication tolerance^5^,^6^. During the acousto-optic interaction process, the launched acoustic waves cause the geometrical deformation and refractive index change, leading to the establishment of dynamic grating^14^,^15^,^16^. The traveling optical mode experiences the reflection arising from the dynamic grating, and a new optical mode is generated under the phase-matched condition. As a result, the mode conversion is fulfilled. Phoxonic crystals have been employed for controlling both the optical and acoustic fields to enhance the acousto-optic interaction^17^,^18^,^19^. Informed by the understanding of the A-O effect and phoxonic crystals, a nanoscale waveguide is thought to realize the on-chip mode conversion for on-chip integration.

In this paper, we propose an on-chip scheme for realizing reversible and tunable optical mode conversion based on the acousto-optic effect in a hybrid phononic-photonic waveguide, and develop a theoretical model to analyze the operating principle of the mode converter. During the mode conversion, the output optical modes can be chosen by adjusting the acoustic frequency and amplitude.

## Results

### The hybrid photonic-phononic waveguide

**Figure 1:**
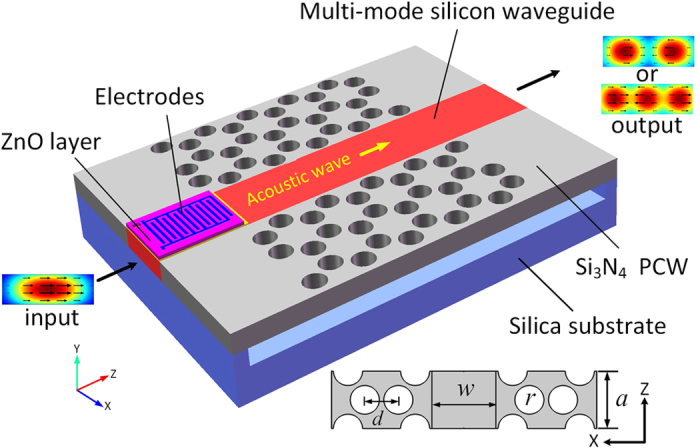
Schematic diagram of the hybrid phononic-photonic waveguide.

Figure 1 depicts the hybrid photonic-phononic waveguide for the proposed mode converter. The hybrid waveguide is suspended in air over the silica substrate. In the hybrid waveguide, the z-direction rectangular silicon (Si) waveguide is embedded in silicon nitride (Si_3_N_4_) slab perforated by a honeycomb lattice of circular holes, and designed for supporting the following the x-polarization optical modes, 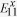, 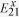 and 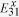, in 1550 nm communication band. The Si waveguide not only serve as optical waveguide to confine the optical modes but also is introduced as a line defect in the Si_3_N_4_ honeycomb phononic crystal to form a phononic crystal waveguide (PCW), which will confine the acoustic wave in the hybrid waveguide. A honeycomb-lattice of circular in the Si_3_N_4_ slab gives rise to phononic bandgaps for guided acoustic modes^19^. It is expected that the absence of radiative modes in the silicon waveguide will reduce the acoustic propagation loss in the hybrid waveguide. The reason is that the silicon waveguide is served as line defect in phononic crystal waveguide, which can only support the guided acoustic mode. Electroacoustic transducer consisted by ZnO layer and electrodes is placed on the Si layer for feeding the acoustic wave into the waveguide. The typical geometry parameters are as follows: the hybrid waveguide with the following parameters, 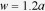 (Si waveguide width), 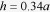 (waveguide thickness), 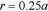 (radius of the holes) and 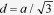, where 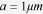 is the lattice period.

### The principle and theoretical model of the mode converter based on dynamic grating

**Figure 2:**
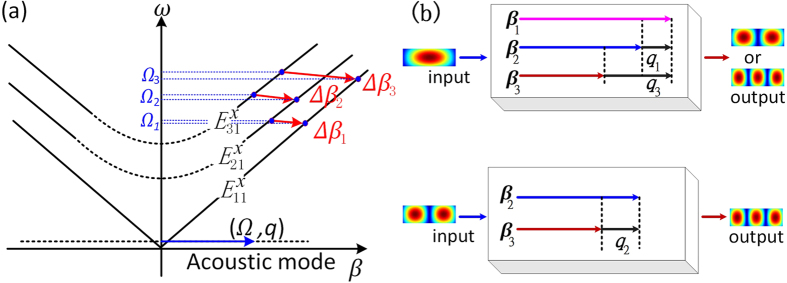
(**a**) Dispersion diagram of the optical and acoustic modes. (**b**) Different phase-matched conditions required in three kinds of mode conversions.

Utilizing the electroacoustic transducer, the longitudinal acoustic wave with frequency at 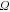 is launched into the hybrid waveguide, which leads to the establishment of dynamic grating for driving the resonant coupling between different optical modes. In this process the energy and momentum conservation require that 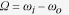 and 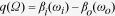, respectively. Here, 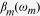 represents the propagation constant and 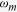 is the optical frequency, with *m= i, o* specifying the input and output optical modes. The 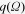 represents the longitudinal acoustic dispersion relationship. Figure 2(a) shows the optical wave vector mismatches (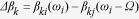, *k* *=* *1, 2, 3*) between different optical modes and the dispersion diagram in the hybrid waveguide. The strong photon-phonon coupling occurs under the phase-matched condition 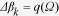 satisfied. Vector representations of phase-matched conditions are shown in Fig. 2(b) in the different mode conversions.

For a waveguide oriented along the z-axis, the electric fields of each mode can be expressed as


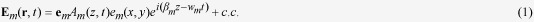


where the 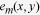 represents the transverse electric field with frequency 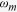 and propagation constant 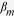. 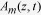 is an envelope function, and 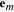 denotes the polarization direction of electric field. In the mode conversion between different optical modes with same polarization, the longitudinal acoustic wave modulates the refractive index of waveguide for forming a dynamic grating^5^. The longitudinal displacement components induced by acoustic wave can written as^15^


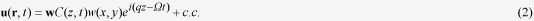


where 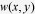 denotes the displacement distribution in x-y plane with the frequency 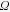 and propagation constant *q*, *C* describes the acoustic field envelope along the waveguide and **w** denotes the displacement direction. The displacement field obeys the acoustic wave equation^20^


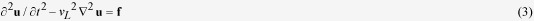


where 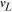 is the longitudinal velocity of acoustic field in the waveguide material, and the electrostrictive pressure per unit volume 
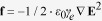
 is given in ref. 12. Where 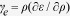 is known as the electrostrictive constant^21^ and 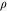 is the material density. 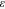 and 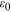
are the relative permittivity and the permittivity contrast of free space, respectively. In additional, the interior air region is modeled using zero-traction boundary conditions at the air-material interface^22^.

Then we analyze how the acoustic wave drive the optical mode conversion. The spatial evolution of the optical fields is described by the wave equation.


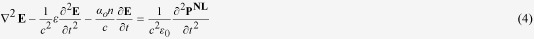


where c is the light velocity of free space, 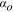 is the optical absorption coefficient and 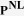 is the nonlinear polarization. The acoustic wave in the hybrid waveguide induces a periodic refractive index perturbation. The longitudinal strain distribution induced by the elasto-optic effect also leads to a geometrical deformation and a change in the refractive index. Both contributions are taken into account in the nonlinear polarization^23^


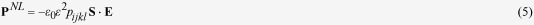


where 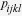 is the elasto-optic tensor and **S** is the strain distribution given by 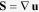. From the Eqs. (1)-(5), the coupled-mode equations for mode conversion process can be obtained under the slowly varying envelope approximation.


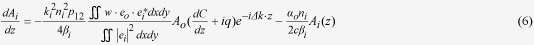



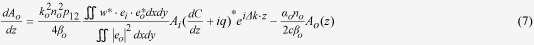



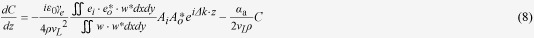


where 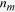
is the effective index, and 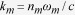. *c* is the speed of light in vacuum, and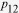 is the elasto-optic coefficient (for silicon 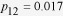). 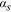 corresponds to the acoustic losses. 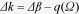 denotes the phase-mismatched between the optical and acoustic waves. Here, the Eqs. (6)-(7,(8) describe the amplitudes of optical and acoustic waves variation along the z-direction in the acousto-optic interaction process in the hybrid waveguide, respectively. Next, we use the developed model to analyze the modal coupling induced by the acousto-optic effect.

### Operation performance of the mode converter

**Figure 3:**
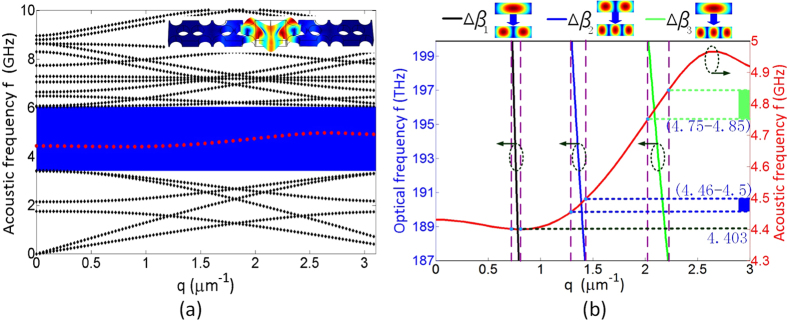
(**a**) Phononic band structure for phononic crystal slab with a = 1 μ*m*^*−*1^ and phononic dispersion curve of the phononic crystal waveguide. Inset is displacement field pattern of the phononic mode (**b**) The acoustic dispersion relationship (red curve) and the propagation constant differences between the different guided optical modes.

Figure 3(a) illustrates the dispersion characteristics of the longitudinal acoustic guided mode (eigenmode). It can be noted that there is an acoustic eigenmode (red dots) appearing inside the bandgap (blue region), and the frequency of the acoustic eigenmode is within the range of 4.4 to 4.96 GHz. The displacement field of the acoustic mode guided by the PCW is shown in inset, which demonstrates that the acoustic guided mode is confined in the Si waveguide. The good overlap between the acoustic and the optical fields manifest a strong optical mode coupling. In the hybrid waveguide, through adjusting the acoustic frequency, the acoustic wave is able to promote the mode conversion of the fundamental mode (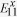) to higher-order modes 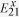, 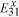 and between 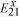 and 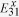. Because the frequency of the acoustic eigenmode is far less than optical frequency, it can be assumed that the optical frequency mismatch is 4.5 GHz between the optical modes in the mode conversions. In this condition, the optical wave vector mismatches in the three kinds of mode conversions, 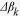 with 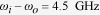, are plotted atop the acoustic dispersion curve (red curve), shown in Fig. 3(b).

The wave vector mismatch value of 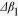 (black curve) between the 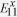-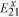 cover from 0.732 to 0.794 μ*m*^−1^ with the optical frequency changing from 187 to 200 THz. Simultaneously, the propagation constant *q* of the injected acoustic wave should be equal to the optical wave vector mismatch for realizing high efficient mode conversion between the 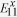 and 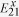. Through the acoustic dispersion curve, the corresponding frequency of the phase-matched acoustic wave (0.732 μ*m*^−1^ < *q* < 0.794 μ*m*^*−*1^) is approximately 4.403 GHz. In the same optical frequency range, the similar conclusion can be drawn that the acoustic frequencies which are used to promote the mode conversion between the 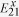-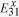 and the 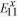-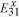 are in the ranges of 4.46 ~ 4.5 GHz and 4.75 ~ 4.85 GHz, respectively.

**Figure 4:**
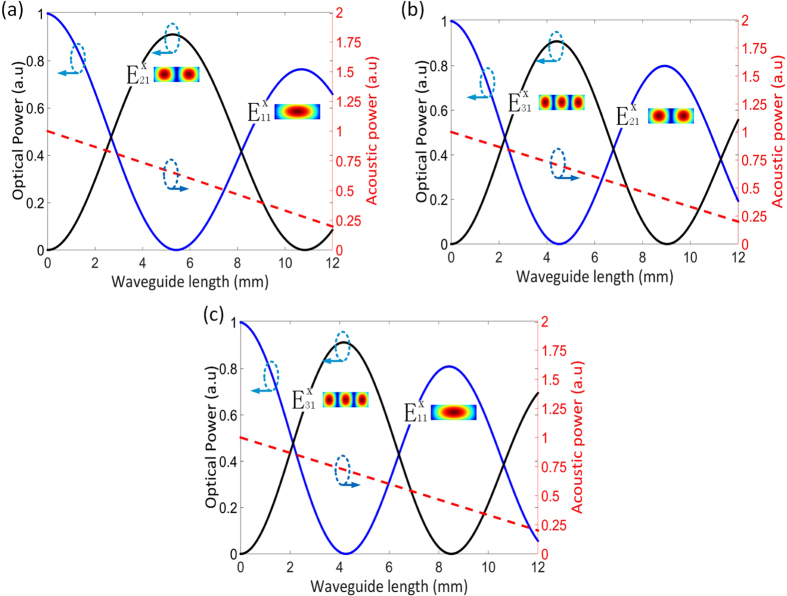
The optical and acoustic powers vary with the hybrid waveguide length in: (**a**) the mode conversion between 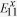
−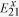, (**b**) the mode conversion between 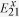
−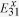, (**c**) the mode conversion between 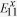
−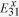.

After we obtain the phase-matched condition (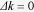) in the hybrid waveguide, the mode conversion can be numerically simulated by solving the coupled-mode equations. In our simulations, the 192 THz optical wave is chosen as the input signal light. We start off with equal optical and acoustic powers, injecting the hybrid waveguide at z = 0 with 5 mW. In addition, It is assumed that the optical loss 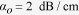 and the mechanical quality factor of Q = 1000 which decides the acoustic loss. For each mode conversion, we calculate the optical and acoustic mode profiles, and then perform the numerical integration to calculate the overlap area. Finally, the variations of optical powers *P*_*m*_(z) and acoustic power *P*_*a*_(z) along the propagation direction z in the three mode conversions are obtained by the calculation of Eqs. (5)-(7), as shown in Fig. 4.

The obtained results show that the variations of the powers are the oscillation processes. At the beginning, an input signal photon translates into another phase-matched mode photon under the effect of dynamic grating established by input acoustic wave. Due to conversion from lower-order optical mode (high frequency) to higher-order mode (low frequency), the same phonon as we inject is created in the process, and the conversion will continue until the input signal photons are consumed completely. The phonons propagate along the hybrid waveguide with low loss resulting from the confinement of PCW. And then, the propagating phonons will drive the reverse mode conversion until the higher-order mode power decreases to zero. The length of the waveguide required by a complete conversion from one optical mode to another mode is called as an efficient length (*L*_*eff*_). Resulting from the difference in overlap area of the acoustic and optical fields, the *L*_*eff*_are clearly distinct in the three mode conversions, as shown in Fig. 4. The *L*_*eff*_ required in mode conversion between 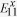-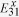 is the shortest with 4.1 mm resulting from the largest overlap between them among the optical modes.

**Figure 5:**
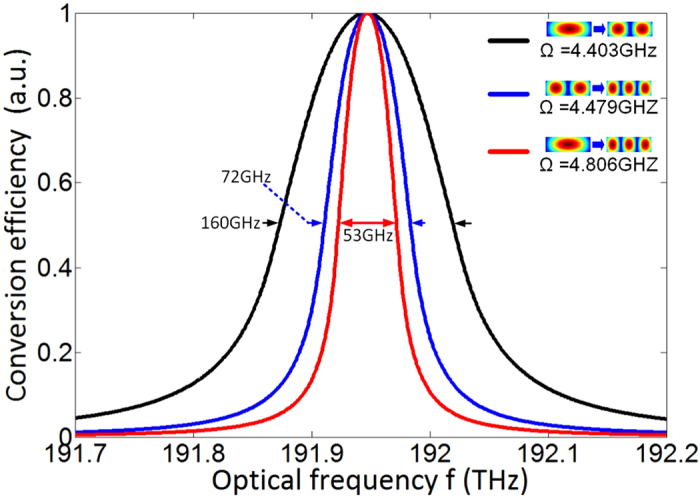
Spectral response in each mode conversion at given acoustic frequencies.

Figure 5 shows the spectral response of each mode conversion in the mode converter obtained by fixing an acoustic frequency in corresponding phase-matched range. At an acoustic frequency of 4.403 GHz, mode conversion between 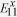 -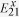 occurred at 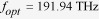 with a 3-dB bandwidth of 160 GHz. For the comparison of bandwidth between different mode conversions at the given acoustic frequency, we choose the other acoustic frequencies with 4.479 GHz and 4.806 GHz to realize the mode conversion between 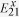-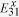 and 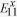 -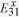 at same optical frequency, respectively. Here, the corresponding 3-dB bandwidths are 72 GHz and 53 GHz, respectively. The bandwidth difference comes from the difference of the slope of acoustic dispersion curve in the three phase-matched ranges in Fig. 3(b).

**Figure 6:**
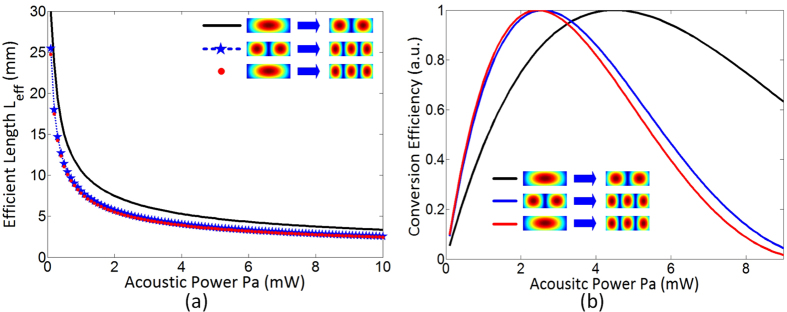
(**a**) Mode conversion efficiency varies with acoustic power under a given waveguide length. (**b**) Efficient lengths in the mode conversions affected by acoustic power.

Figure 6(a) displays the influences of input acoustic power on the *L*_*eff*_ in the three kinds of mode conversions with the fixed optical power at 5 mW. When the input acoustic power is lower than 2 mW, the *L*_*eff*_ decreases exponentially with the increase of acoustic power. As the acoustic power continues to increase, the rate of deceleration of *L*_*eff*_ will be weak. This implies that there is a proper acoustic power under the suitable waveguide length in each mode conversion. Nonetheless, the optimal acoustic power required in different mode conversions is obvious in a same waveguide. Based on this evidence, Fig. 6(b) shows the mode conversion efficiency as a function of the acoustic power with the waveguide length at 5 cm in the mode conversions. The acoustic wave amplitude determines the modulation depth of the refractive index to affect the conversion efficiency. When the input acoustic power is smaller than optimal value, the conversion efficiency grows rapidly with the acoustic power increase. As the acoustic power reaches to optimal value, the hybrid waveguide can realize the complete optical mode conversion. If we continue to increase the acoustic power, the conversion efficiency will reduce due to the reverse mode conversion. Therefore, we can achieve the optimal mode conversion efficiency for each mode conversion in the same hybrid waveguide by adjusting the acoustic power, which is significant for the hybrid waveguide fabrication and integration.

## Discussion

In this work, we propose a hybrid photonic-phononic waveguide to realize the reversible and tunable optical mode conversion based on the dynamic grating. The theoretical model and simulation of the acousto-optic effect are presented to illustrate the mode conversion over a large bandwidth in the hybrid waveguide. The hybrid waveguide enables independent control of optical and acoustic wave to enhance the acousto-optic interaction. The acoustic wave amplitude determines the mode conversion efficiency in a fixed-length waveguide. Furthermore, we obtain three different mode conversions by choosing the different acoustic wave frequency. It is predicted that the more kinds of mode conversions can be obtained by designing a suitable hybrid waveguide based on the acousto-optic effect. A chip-based tunable mode converter scheme with a large operation bandwidth will play an important role in modern integrated photonics.

## Methods

To obtain the dispersion characteristics of the PCW, the finite element package COMSOL together with the appropriate boundary conditions is utilized in our simulation. As the defect is introduced, the super-cell dimensions have been properly chosen to avoid the interaction between neighboring waveguides.

## Author Contributions

J.Q.S. conceived the study. G.D.C. and R.W.Z. performed the numerical simulation. G.D.C. analyzed the data and wrote the manuscript. J.Q.S. supervised the project and edited the manuscript. All authors discussed the results and commented on the manuscript.

## Additional Information

**How to cite this article**: Chen, G. *et al*. On-chip optical mode conversion based on dynamic grating in photonic-phononic hybrid waveguide. *Sci. Rep.* **5**, 10346; doi: 10.1038/srep10346 (2015).
